# The role of NGOs in rural Vietnam: a case study and critique

**DOI:** 10.1186/1753-6561-6-S4-P54

**Published:** 2012-07-09

**Authors:** Y Barzin

**Affiliations:** 1 Brighton & Sussex Medical School, UK

## Introduction

There is on-going debate about the merits of programmes supported by international aid, with critics arguing that many programmes are unsustainable, that they follow international agendas and lead to fragmentation of national health systems. The debate focuses on large providing for patients' health needs and the impact of the programme within the public health system.

Vietnam has good health status compared to countries with similar economies, which can largely be attributed to its focus on primary care and the extensive coverage by commune health centres. Yet, rural populations have limited access to quality services. Vietnam faces a double burden of chronic and communicable diseases.

## Methods

An ethnographic study was conducted on a charity's medical programme, which provides free temporary clinics for rural populations in central Vietnam. Data from medical notes was collected and interviews with patients and professionals were conducted.

## Results

It is likely that a significant proportion of patients were covered by Vietnam's social insurance scheme; however high costs and poor resources at public clinics limit access to quality care. The majority of diagnoses in clinics were for chronic problems, correlating with increasing age, which the programme is not designed to manage. The benefits of consultations were further limited by poor patient health literacy and lack of time and resources. The charity works closely with local authorities and programmes are developed at their request. The clinics do not focus on specific diseases and reflect the horizontal approach of public clinics. However, the clinics duplicate existing services and do not contribute to development of the public health system.

## Conclusions

Despite its limitations, the programme can be beneficial to the local communities. Of importance, the programme is no seen to undermine the public health system. However, the programme cannot meet the needs of rural communities as the majority suffer from chronic illness. This study highlights the need to consider programme-specific factors within the debate on international aid.

